# Hospital quality measures: are process indicators associated with hospital standardized mortality ratios in French acute care hospitals?

**DOI:** 10.1186/s12913-017-2534-3

**Published:** 2017-08-22

**Authors:** Marcus Ngantcha, Marie-Annick Le-Pogam, Sophie Calmus, Catherine Grenier, Isabelle Evrard, Agathe Lamarche-Vadel, Grégoire Rey

**Affiliations:** 1 Inserm, CépiDc (Epidemiology center on medical causes of death), Kremlin-Bicêtre, France; 2 Institute of Social and Preventive Medicine (IUMSP), Lausanne University Hospital (CHUV), Lausanne University (UNIL), Lausanne, Switzerland; 3 Haute Autorité de santé (HAS), Service des indicateurs pour l’amélioration de la qualité et de la sécurité des Soins (SIPAQSS), Saint-Denis La Plaine, France; 4 Université Paris Sud, Kremlin-Bicêtre, France

**Keywords:** Quality of care, Hospital Process Indicators, Hospital Standardized Mortality Ratio, SIMEX method

## Abstract

**Background:**

Results of associations between process and mortality indicators, both used for the external assessment of hospital care quality or public reporting, differ strongly across studies. However, most of those studies were conducted in North America or United Kingdom. Providing new evidence based on French data could fuel the international debate on quality of care indicators and help inform French policy-makers. The objective of our study was to explore whether optimal care delivery in French hospitals as assessed by their Hospital Process Indicators (HPIs) is associated with low Hospital Standardized Mortality Ratios (HSMRs).

**Methods:**

The French National Authority for Health (HAS) routinely collects for each hospital located in France, a set of mandatory HPIs. Five HPIs were selected among the process indicators collected by the HAS in 2009. They were measured using random samples of 60 to 80 medical records from inpatients admitted between January 1st, 2009 and December 31, 2009 in respect with some selection criteria. HSMRs were estimated at 30, 60 and 90 days post-admission (dpa) using administrative health data extracted from the national health insurance information system (SNIIR-AM) which covers 77% of the French population. Associations between HPIs and HSMRs were assessed by Poisson regression models corrected for measurement errors with a simulation-extrapolation (SIMEX) method.

**Results:**

Most associations studied were not statistically significant. Only two process indicators were found associated with HSMRs. Completeness and quality of anesthetic records was negatively associated with 30 dpa HSMR (0.72 [0.52–0.99]). Early detection of nutritional disorders was negatively associated with all HSMRs: 30 dpa HSMR (0.71 [0.54–0.95]), 60 dpa HSMR (0.51 [0.39–0.67]) and 90 dpa HSMR (0.52 [0.40–0.68]).

**Conclusion:**

In absence of gold standard of quality of care measurement, the limited number of associations suggested to drive in-depth improvements in order to better determine associations between process and mortality indicators. A smart utilization of both process and outcomes indicators is mandatory to capture aspects of the hospital quality of care complexity.

**Electronic supplementary material:**

The online version of this article (doi:10.1186/s12913-017-2534-3) contains supplementary material, which is available to authorized users.

## Background

In recent decades, measuring quality of care in hospitals has become a major challenge for many countries. Indeed, measuring is crucial for assessing and improving internal quality, informing health policies and justifying patients’ choices. It is also requested by payers for performance assessment and value-based purchasing [1]. Three types of measures are commonly used to assess the quality of care in hospitals: structural, process and outcome indicators [2–5]. Structural measures relate to the characteristics of the health-care setting [3] such as the number of units and equipment, number and qualifications of medical and nursing staff, etc. Assessing an association between structural measures and both outcomes and process indicators may sometimes be very tricky. Indeed, increasing health resources does not necessarily lead either to a reduction in mortality or an improvement in processes [5]. Process indicators (PIs) aim to assess the quality of clinical processes and answer the following question: do patients receive the best care possible according to current knowledge? This implies that achieving the best care processes leads to better health [6]. Thus, PIs have to be strongly associated with related health outcomes to be used for quality assessment. Apart from this requirement, PIs present many advantages and are commonly used in quality improvement, public reporting (e.g. Hospital Inpatient Quality Reporting Program in the United States or the Canadian Institute for Health Information’s hospital performance program), or pay-for-performance (e.g. Medicare Hospital Quality Alliance Program [7]) programs [8]. Furthermore, they may be used for hospital accreditation [9, 10]. Outcome measures of hospital care quality generally refer to patient health status as a result of health-care processes or to patient experience with hospital care. Among outcome indicators, mortality rates are the most widespread measures. They answer the question about inpatients’ survival or death during a fixed or variable period of time. Indeed, mortality is typically the type of information that the public and patients are interested in [3, 5]. It is easily measurable, understandable to everyone, and supposedly cheaper to produce than other types of indicators since it is regularly collected in countries with health information systems. Moreover, it is frequently used for comparing performance between hospitals [11]. However, like other outcome indicators, mortality rates depend on various factors including patient case-mix [12] (i.e. patient characteristics, comorbidity and severity at admission) and data accuracy [13], which could be confounding factors for measuring quality of care. Hence, they have to be accurately measured and adjusted on case-mix before comparing mortality across providers or being used for hospital profiling. Owing to the above mentioned limitations, they act as signals or flags to identify structures where further investigations have to be conducted [13].

Many studies aiming to assess the relationship between process and mortality indicators have been conducted mainly on US and UK data [7, 11, 14–17]. Various statistical methods have been used to assess this relationship ranging from simple correlations to hierarchical models. The underlying hypothesis is that hospitals with high mortality rates are very likely to have poor PI results [8]. However, some studies have failed to show this association. A systematic review published in 2007 [17] which included 36 studies examining 51 relationships between PIs and risk-adjusted mortality found a positive correlation in only half of the relationships (51%). There was no association between the two types of indicators in 31% of the relationships and paradoxical associations in 18%. The authors concluded that there is neither consistency nor reliability to assert that high risk-adjusted mortality is related to poor quality of care in hospitals. In this context, providing new evidence based on French data would be of significant interest [18–20]. Moreover, our study should fuel the quality-of-care debate in France and help inform French policy-makers’ decision regarding the inclusion of quality indicators into quality improvement, public reporting, pay-for-performance and accreditation programs.

The objective of our study was to explore whether optimal care delivery in French hospitals as assessed by their PIs was associated with low HSMRs measured for different conditions and timeframes.

## Methods

### Hospital Process Indicators (HPIs)

Since 2008, French hospitals have been subjected to an annual collection of mandatory indicators. These indicators have been progressively integrated in the National Quality Improvement Framework driven by the French National Authority for Health (Haute Autorité de Santé, HAS) and including public reporting, hospital accreditation and pay-for-performance.

In 2009, the HAS collected 13 mandatory HPIs designed to evaluate four components of hospital care processes: 1) Inpatient medical records; 2) Anesthesia records; 3) Multidisciplinary team meetings in oncology; 4) In-hospital care for myocardial infarction. In our study, we selected 5 HPIs among the 13 that focused on three priority areas: continuity of care, early detection of nutritional disorders, and adherence to best practice guidelines. Those 5 indicators were a priori selected based on both the public availability of their results and their recording for all patients.

HPI1 (“Early Detection of Nutritional Disorder level 1”) estimates, for each hospital, the proportion of adult inpatients (≥18 years at admission) hospitalized in acute care during the year for whom weight is recorded in medical or nursing notes within 2 days of admission [21]. Weight measurement is a pre-requisite for the successful prevention of nutritional disorders.

HPI2 (“Beta-blocker, Antiplatelet agent, Statin and ACE Inhibitor prescription at discharge in the treatment of acute myocardial infarction”, BASI score) [22] evaluates the appropriate prescription at discharge of four medications recommended for the treatment of inpatients with acute myocardial infarction. It estimates, for each hospital, the proportion of adult inpatients admitted during the year for an acute myocardial infarction and who were prescribed four medications at discharge according to the French professional guidelines.

HPI3 (“Multidisciplinary Team (MDT) Meetings in Oncology”) estimates, for each hospital, the proportion of inpatients of all ages at admission, hospitalized for an initial cancer treatment during the year, and for whom a MDT meeting is recorded in their medical notes. MDT meetings have become mandatory since 2007 in France and must be mentioned in the patient’s medical records [23].

HPI4 (“Completeness and Quality of Anesthetic Records”) [21] assesses the appropriate reporting of anesthetic information in inpatient medical records. Required information in medical records is audited using 13 criteria (if applicable) including six for the pre-anesthetic period, two for the per-anesthetic period, three for the post-anesthetic period, and one encompassing the three periods. For each acute care hospital, individual proportions of fulfilled criteria among the 13 (if applicable) are calculated for randomly selected inpatients of any age, who underwent general or regional anesthesia during a surgical procedure. HPI4 is then calculated as the average of individual proportions among randomly selected inpatients during the year of interest. Appropriate reporting of anesthetic information contributes to care coordination and anesthetic risk control in patients admitted to surgical units.

HPI5 (“Completeness and Quality of Medical Records”) assesses the appropriate reporting of ten items in inpatients’ medical notes [23]. It is the most important indicator for assessing care coordination. Indeed, medical records are the main tool for sharing patient information and ensuring continuity of care. Required information in medical records is audited using the ten criteria (if applicable). For each acute care hospital, individual proportions of fulfilled criteria among the ten (if applicable) are calculated for randomly selected inpatients of any age hospitalized in acute care. HPI5 is then calculated as the average of individual proportions among randomly selected inpatients during the year of interest.

HPIs were estimated for each hospital by auditing samples of inpatient medical records randomly selected between January 1st, 2009 and December 31st, 2009. Medical records were selected according to inclusion and exclusion criteria specific to each HPI denominator, as defined by HAS expert groups (see selection criteria in Additional file 1). HAS experts determined the acceptable sample size needed to estimate each HPI: 60 for HPI2, HPI3 and HPI4 and 80 for HPI1 and HPI5 [24, 25]. This choice of 60 to 80 records resulted from a trade-off between indicator precision or statistical power in between-hospital comparisons, and the workload generated by this data collection within each hospital [24].

### Hospital Outcome Indicators (HOIs)

In 2010, the “Post-Hospital Mortality Analysis, aiming at estimating Indicators” project (AMPHI) was conducted in France to assess the feasibility of providing measures of in- and post-hospital mortality using administrative health data [18, 19]. This project led to the development of a French version of the hospital standardized mortality ratio (HSMR) [19] based on 2009 civil year data (1st January to 31st December). These data were obtained by individual linkage of two nation-wide databases including the national hospital discharge database for acute care hospitals (PMSI-MCO) and the national health insurance claims database (SNIIR-AM). PMSI-MCO contains medical and administrative information on all hospital stays for all acute care hospital categories (i.e. public, private for profit, private non-profit hospitals). In particular, it also includes length of stay, provenance, discharge destination, main diagnosis, secondary diagnoses (coded according to the 10th revision of the international Classification of Diseases and related health problems, ICD-10) and procedure codes (coded according to the French classification for procedures, CCAM). SNIIR-AM comprises, for the whole French population, individual information on all ambulatory care services reimbursed by the French national health insurance. It also contains vital status and dates of death for 77% of the French population insured under the general health insurance scheme in 2009 [26]. PMSI-MCO and SNIIR-AM have been routinely linked at patient level since 2007. Consequently, it was possible to count deaths at specific timeframes after admission (30, 60 and 90 days post-admission (dpa)).

HSMRs were calculated with an updated method developed in the UK by the Dr. Foster Unit at Imperial College London [20, 27]. This methodology was adapted notably to cover 100% stays [28]. The main diagnoses were assigned to the clinical classification system (CCS) categories (**http://www.hcup-us.ahrq.gov/toolssoftware/ccs/ccs.jsp**). For each hospital, its expected number of deaths was considered as the sum of predicted risks of death over all stays. The latter was obtained by logistic regression for each CCS or group of CCSs (for CCSs within the lower quintile of mortality risk) adjusted for available case-mix factors. The factors included were: age (fractional polynomial [29]), deprivation [30], sex, Charlson comorbidity index [31] based on secondary diagnoses (recalibrated on French data [27]), an interaction term between Charlson index and age, month of admission, source of admission and subcategory diagnostic group. The full description and specificities of our methodology can be found elsewhere [19].

The whole process was repeated for each selected timeframe (30, 60 and 90 dpa) and for each HPI (specific HSMR). Indeed, for each HPI, hospital stays participating in the related HSMR estimation were selected according to the defined inclusion and exclusion criteria of the HPI denominator [see Additional file 1]. Given that some hospitals did not have any surgery department or cancer treatment unit or did not deliver acute myocardial infarction treatment, numbers of hospitals involved in HPI calculation were different across HPIs. Hospitals finally involved in this analysis were restricted to those having the same identification number (FINESS number) for process and outcome measures.

### Statistical analysis

Assessing the association between HPIs and specific HSMRs requires in-depth knowledge about their distribution. The HSMR numerator, namely the observed number of deaths, was assumed to follow a Poisson distribution. Given that mortality is commonly used as an ultimate outcome, HSMRs were chosen as the dependent variable. A Poisson model was thus built with HPIs as the explanatory variables. The convenient but relatively small sample sizes to estimate HPIs introduce measurement errors [32].

Several methods enable to correct this error under certain hypotheses [32]. One of these, the simulation extrapolation method (SIMEX), was applied to correct for measurement error in HPI estimation, under the hypothesis of a classical additive error [32]. Thus, for each specific HSMR, we considered log-linear regression model of Y (i.e. HSMR) on X (HPI) where predictor X cannot be observed as it is affected by a measurement error. Assuming that the measurement error is additive means that instead of X, we observe W = X + U where U ~ Normal (0, σ^2^u). In addition, we assume that U is independent of X and Y. Consequently, if U equals 0, there is no error in the measurement of X [33]. SIMEX simulates contaminated datasets (i.e. datasets of W values with increasingly larger amounts of measurement errors) in order to study the effect of measurement error on the fitted coefficients. A corrected coefficient is then extrapolated from the simulated ones and its precision is estimated using asymptotic or re-sampling methods. The full description of this method can be found in Additional file 2 in supplementary materials.

Secondary analyses were performed to evidence correlations between HSMRs and HPIs without correcting for measurement error. We used weighted Pearson correlation tests when HPIs were likely to follow a normal distribution. When this was not the case, a Spearman correlation test was conducted. The significance level for the entire tests was 0.05 in bilateral formulation. Adjustment for the first type risk error was not considered due to the exploratory purpose of this study [34].

The analyses were conducted with SAS® software except for the SIMEX method which was performed using R version 3.1.1 with the SIMEX package (version 1.5).

## Results

HSMRs were calculated for 1284 hospitals located in France based on the 2009 acute care discharge database (PMSI-MCO) which comprised 11,526,545 stays (Table 1). HSMR medians were close to 1 and the inter-quartile range decreased for longer timeframes which means that observed and expected mortality tends to be similar as the time since admission increases. The lowest median of the HSMR based on the HPI2 denominator population was the furthest from 1 (from 0.30 for 30 dpa to 0.72 for 90 dpa) and was estimated on the smallest number of hospitals (304).

**Table 1:** Distributions of Hospital Standardized Mortality Ratios for the five inpatient populations included in HPIs’ denominators

HSMRs	Global (*N* = 1284)		Specific to HPI1 (*N* = 1064)		Specific to HPI2 (*N* = 304)		Specific to HPI3 (*N* = 730)		Specific to HPI4 (*N* = 949)		Specific to HPI5 (*N* = 1068)	
	Median	IQR	Median	IQR	Median	IQR	Median	IQR	Median	IQR	Median	IQR
30 dpa	0.94	[0.83–1.13]	1.00	[0.90–1.16]	0.30	[0.00–1.29]	0.91	[0.74–1.10]	1.01	[0.88–1.17]	0.97	[0.87–1.09]
60 dpa	0.96	[0.86–1.10]	0.97	[0.89–1.10]	0.62	[0.00–1.19]	0.94	[0.81–1.08]	0.99	[0.86–1.12]	0.97	[0.90–1.09]
90 dpa	0.97	[0.89–1.09]	0.98	[0.90–1.08]	0.72	[0.25–1.17]	0.96	[0.83–1.08]	1.00	[0.87–1.10]	0.97	[0.90–1.08]

*HPI* Hospital Process indicators, *HPI 1* Nutritional Disorders, *HPI 2* Beta-blockers. Antiplatelet agent. Statin and ace Inhibitor, *HPI 3* Multidisciplinary Team Meetings in management, *HPI 4* Completeness and Quality for Anesthetic Records, *HPI 5* Completeness and Quality of Medical Reports contents, *HSMR* Hospital Standardized Mortality Ratio, *dpa* day post admission

HPI results and numbers of hospitals evaluated using HPI’s in 2009 are described in Table 2. Standard deviations and inter-quartile ranges were large compared to means and medians, somewhat reflecting the between-hospital variation. For example, the HPI3 mean was 37.70 and its standard deviation was 31.93.

**Table 2:** Description of Hospital Process Indicators

Name of HPI	Number of hospitals	Mean	STD	Coefficient of variation	Median	IQR
HPI1	1064	74.86	21.00	0.28	80.00	[62.5–92.5]
HPI2	304	70.82	20.42	0.29	73.51	[59.41–86.66]
HPI3	730	37.70	31.93	0.85	36.67	[3.33–66.00]
HPI4	949	75.04	13.18	0.18	75.67	[66.51–84.36]
HPI5	1068	69.84	11.38	0.16	69.39	[62.11–77.02]

*HPI* Hospital Process indicators, *HPI 1* Nutritional Disorders, *HPI 2* Beta-blockers. Antiplatelet agent. Statin and ace Inhibitor, *HPI 3* Multidisciplinary Team Meetings in management, *HPI 4* Completeness and Quality for Anesthetic Records, *HPI 5* Completeness and Quality of Medical Reports contents, *HSMR* Hospital Standardized Mortality Ratio, *STD* standard error, *IQR* inter-quartile interval

The results of Poisson models corrected with the SIMEX method are presented in Table 3. HPI4 was negatively associated with the short timeframe 30 dpa HSMR (0.72 [0.52–0.99]) but not with 60 dpa or 90 dpa HSMRs. This means that there was a 28% reduction in 30 dpa HSMR for an HPI4 score changing from 0 to 1 (0 to 100 for percentage). HPI1 was negatively associated with all HSMRs: 30 dpa HSMR (0.71 [0.54–0.95]), 60 dpa HSMR (0.51 [0.39–0.67]) and 90 dpa HSMR (0.52 [0.40–0.68]). HPI2, HPI3 and HPI5 were not associated with any HSMR whatever the timeframe.

**Table 3:** Associations between HPIs and HSMRs (using SIMEX method)

HPI	HSMR					
	30 dpa		60 dpa		90 dpa	
	RR	IC	RR	IC	RR	IC
HPI1	0.71	[0.54; 0.95]*	0.51	[0.39; 0.67]*	0.52	[0.40; 0.68]*
HPI2	3.9	[0.83; 18.30]	3.17	[0.90; 11.13]	1.31	[0.35; 4.85]
HPI3	0.93	[0.70; 1.24]	1.02	[0.74; 1.39]	0.93	[0.76; 1.13]
HPI4	0.72	[0.52; 0.99]*	0.83	[0.61; 1.13]	0.86	[0.67; 1.11]
HPI5	0.88	[0.69; 1.12]	0.9	[0.73; 1.11]	0.88	[0.73; 1.07]

HPI: Hospital Process indicators; HPI 1: Nutritional Disorders; HPI 2: Beta-blockers. Antiplatelet agent. Statin and ace Inhibitor; HPI 3: Multidisciplinary Team Meetings in management; HPI 4: Completeness and Quality for Anesthetic Records; HPI 5: Completeness and Quality of Medical Reports contents; HSMR: Hospital Standardized Mortality Ratio

*Results with *p* < 0.05 are significant

When testing correlations between HSMRs and HPIs without correcting for measurement error, we evidenced that HPI1 was inversely associated with the three HSMRs: −0.19 (30 dpa HSMR), −0.24 (60 dpa HSMR) and −0.26 (90 dpa HSMR) (Table 4). Unlike analyses using the SIMEX approach, HPI3 was inversely associated with the three HSMRs: 30 dpa HSMR (−0.12), 60 dpa HSMR (−0.15) and 90 dpa HSMR (−0.15). Furthermore, HPI4 was inversely associated with the HSMRs: 30 dpa HSMR (−0.07), 60 dpa HSMR (−0.07) and 90 dpa HSMR (−0.08).

**Table 4:** Correlations between HSMRs and HPIs without correcting for measurement error

HPI	HSMR					
	30 dpa		60 dpa		90 dpa	
	Corr	*p*-value	Corr	*p*-value	Corr	*p*-value
HPI1	−0.19*	<.0001	−0.24*	< .0001	−0.26*	< .0001
HPI2	0.10	0.07	0.1	0.06	0.07	0.22
HPI3	−0.12*	0.001	−0.15*	< .0001	−0.15*	<.0001
HPI4	−0.07*	0.03	−0.07*	0.02	−0.08*	0.01
HPI5	−0.04	0.18	−0.05	0.11	−0.04	0.18

*Results with *p* < 0.05 are significant

## Discussion

This study showed significant associations between HSMRs and some HPIs, which strengthens the idea that low hospital mortality may be associated with high-level of process indicators. Indeed, HPI1 was negatively associated with all HSMRs: 30 dpa HSMR (0.71 [0.54–0.95]), 60 dpa HSMR (0.51 [0.39–0.67]) and 90 dpa HSMR (0.52 [0.40–0.68]). Also, HPI4 was negatively associated with 30 dpa HSMR (0.72 [0.52–0.99]). This result is at the limit of statistical significance, but anesthetic record contributes to the sharing of information between different physicians involved in every step of the anesthetic management. This information is thus a necessary element in the coordination of care and contributes to the control of the anesthetic risk. Nevertheless, several associations were not statistically significant, particularly for BASI score (HPI2), MDT meetings in oncology (HPI3) and completeness and quality of medical records (HPI5). The limited number of significant and expected associations obtained after the corrections of measurement errors is consistent with the results of a systematic review published by Pitches et al. [17]. Considering this lack of associations among extended number of conditions studied, the public reporting of health care quality indicators may be questionable. Indeed, the risk of institutional stigma drove by such indicators should be avoided [35].

### Strengths of the study

The main strength of our study is the methodological approach developed to assess the association between HSMRs and HPIs. To our knowledge, this is the first time that a method correcting for measurement error has been used to improve the accuracy of association estimates between outcome and process indicators. To underline this, secondary analyses performed without any corrections of HPI measurement error revealed eight supplementary associations (HPI3 and HPI4). While these associations seem interesting and conceptually conceivable, the lack of correction in HPI measurement error means that no conclusions should be drawn. Therefore, we recommend using measurement error correction methods, as it is more adapted to the present design, and it critically impacts the results.

There is still a debate about the suitable timeframes [36, 37] to assess quality of care using mortality indicators. Several authors suggest selecting specific timeframes for specific diseases. In the absence of a gold standard, we decided to select several post-admission timeframes (from 30 days to 90 days).

Another strength of our study is the broad range of diseases and care settings considered. Unlike other studies focusing on a single condition such as myocardial infarction [14, 38], heart failure [15, 16] and acute coronary syndrome [39], the indicators used are related to several diseases (e.g. myocardial infarction, cancer), settings (e.g. medical or surgical units), and to hospital care management in general.

Finally, the same inclusion and exclusion criteria were applied to select the medical records involved in the calculation of process and outcome indicators, thereby avoiding paradoxical relationships (better process of care associated with higher mortality ratio). Indeed, Pitches et al., stressed that such relationships may be susceptible to ecological fallacy. Indeed, mortality indicators are sometimes measured for entire hospitals while process indicators focus on specific sub-groups of patients within those hospitals. [17].

### Limitations of the study

If a unique dimension of quality of care was consistently assessed both by HPIs and HSMRs, then hospital with high HPIs should have lower HSMRs. The small number of associations in our results raised some concerns about the potential limitations of both HSMRs and HPIs.

The reliability and validity of HSMRs based on French data are not devoid of criticism. Firstly, administrative health data used to measure HSMRs may lead to biased estimations due to the use of different codification strategies across hospitals [20, 40]. Such biases were reported in 2009 by the French Technical Agency of Hospital Information which is especially responsible for the nation-wide collection of hospital discharge data [40]. The improvement in coding rules and the amplification of control procedures by the national health insurance could progressively mitigate these biases. Moreover, despite important changes in coding rules and in the definition of main diagnosis in March 2009, these changes did not greatly affect Charlson index and HSMR calculations [19].

Secondly, according to Dr. Foster Unit’s recommendations, HSMRs were built sequentially: the same data were iteratively used to estimate individual Charlson indexes and HSMRs that are adjusted on Charlson index. It would now be interesting to assess the contribution of an optimal integrated approach to estimate expected mortality and assess the potential modification in the association between HPI and mortality outcomes. Thirdly, the lack of adjustment [41] on important predictors such as the severity of the main condition at admission (information not recorded) in the calculation of HSMRs may have weakened their estimations [13, 42]. Inevitably, the limited number of associations in our findings also raised the question of using other outcomes indicators (e.g. Patient safety indicators) [13]. The preventable deaths when necessary data are collected can also be considered as an alternative outcome for quality of care [43, 44].

For HPIs, firstly, the choice for the number of audited medical records (60 or 80 medical records per hospital) results from a compromise between accuracy of indicators and acceptability regarding the workload of data collection for hospitals. Even though the accuracy obtained is adequate for intended uses of HPIs, the small sample size still induce substantial measurement errors in the context of this study [25].

Secondly, process indicators are based on laws and guidelines. Although guidelines are « evidence based », they do not rely on randomized controlled trials that would demonstrate a causal association between care processes selected as performance measures and mortality measures [45]. Thirdly, inconclusive associations between quality measures on health care processes and mortality could also be explained by non-hospital care-related factors (e.g. socioeconomic context, local healthcare organization) which would affect health outcomes after discharge. These factors (being out of the hospital control will impact the HSMR solely, especially if there is no follow-up after discharge [46]. Furthermore, process indicators are based on traceability of information collected in medical records and may not accurately reflect care delivery [21].

Information recorded in databases for both estimating HSMRs and additional HPIs improved continuously over the past years. Indeed, information is now more accurate, more variables are recorded, and new indicators (e.g. assessing the quality of stroke care or hemodialysis) are under development. These improvements would certainly provide interesting additional results. Furthermore, the use of specific causes mortality as well as other methods of estimations could improve the accuracy of the association. For example, the modeling could be based on two-level hierarchical models (stays and hospital levels). HSMRs could be used as dependent variables while HPIs could serve as independent variables adjusted for hospital characteristics, having then the same value for all stays in the same hospital.

## Conclusion

Given the various dimensions of process quality covered by HPIs and various timeframes involved in HSMRs, this study gives a broad view of the association between process and outcome indicators, especially in French inpatients. Due to the limited number of associations after measurement error correction, our findings warn French policymakers against inappropriate use of both types of indicators: each indicator must be evaluated suitably for each use. Given that any type of indicators solely cannot fully capture the quality of care complexity, comparative assessment between hospitals, public reporting or regulation should be based on a simultaneous utilization of the two types of indicators. New HPIs developed recently together with different outcome indicators, such as preventable death rate, should be assessed to demonstrate how improved processes contribute to improve patient outcomes.

## Additional files

Additional file 1: Inclusion and exclusion criteria for HPI. (DOCX 16 kb)

Additional file 2: Description of the SIMEX method [47–50]. (DOCX 48 kb)

## Abbreviations

AMPHI: Post-Hospital Mortality Analysis, aiming at estimating Indicators
BASI: Beta-blocker, Antiplatelet agent, Statin and ACE Inhibitor prescription at discharge in the treatment of acute myocardial infarction
CCAM: French classification for procedures
CCS: Clinical Classification System
HAS: French National Authority for Health
HPI 1: Nutritional Disorders
HPI 2: Beta-blockers. Antiplatelet agent. Statin and ace Inhibitor
HPI 3: Multidisciplinary Team Meetings in management
HPI 4: Completeness and Quality for Anesthetic Records
HPI 5: Completeness and Quality of Medical Reports contents
HPI: Hospital Process Indicators
HSMRs: Hospital Standardized Mortality Ratios
ICD-10: International Classification of Diseases 10 version
MDT: Multidisciplinary Team Meeting
PIs: Process Indicators
PMSI-MCO: French national hospital discharge database for acute care hospitals
SIMEX: Simulation extrapolation
SNIIR-AM: National Health Insurance Information System
UK: United Kingdom
US: United States
