# Supplementary material for: Hospital quality measures: are process indicators associated with hospital standardized mortality ratios in French acute care hospitals?
Source: BMC Health Serv Res. 2017 Aug 22;17:578. doi: 10.1186/s12913-017-2534-3 (PMC5568353; doi:10.1186/s12913-017-2534-3)
Supplement: Supplementary file 1 — Inclusion and exclusion criteria for HPI. (DOCX 16 kb) [file 12913_2017_2534_MOESM1_ESM.docx]

***Additional file 1:***

**HPI1: Nutritional Disorders Detection - level 1**

Inclusion criteria:

Hospital stays > one day without medical sessions

Exclusion criteria:

Hospital stays with major diagnostic category 28

Hospital stays with day of entry corresponding to day of discharge

Newborns hospital stays “non-hospitalized”

Hospital stays with service inter-establishment

Hospital stays with error in Diagnosis Related Group

Hospital stays with 23K02Z in clinical classification group (night exploration)

Hospital stays for patients < 18 years old

Hospital stays < 2 days

Hospital stays with first two days in accident and emergency department, intensive care unit, continuous surveillance unit (UHCD), maternity, short-term hospitalization unit or palliative care unit.

**HPI2: Beta-blockers, Antiplatelet agent, Statin and ACE Inhibitor prescription at discharge for treating Myocardial Infarction (BASI score)**

Inclusion criteria:

Hospital stays for at least one day (without medical sessions of alive discharged patients with ICD 10 codification corresponding to myocardial infarction as principal diagnosis (codes I21.0x to I21.9x and I22.xx).

Exclusion criteria:

Hospital stays with major diagnostic category 28

Hospital stays with the day of entry corresponding to day of discharge

Hospital stays of patients deceased during hospitalization

Hospital stays with service inter-establishment

Hospital stays where discharge mode is transfer to intensive care unit

Hospital stays with error in Diagnosis Related Group

Hospital stays with medical record unrecovered

Hospital stays with inconsistency in PMSI-MCO

Patient who do not give their consent for exploitation of their medical record

Terminally ill patients in whom therapy withheld in accordance with family’s wishes

**HPI 3: Multidisciplinary Team (MDT) Meetings in oncology**

Inclusion criteria:

Hospitals stay with a principal diagnosis from C00 –C43, C45 – C75, C77 – C79 and C81 – C97 associated with surgical act or radiotherapy during second semester.

Hospital stays with principal diagnosis of Z51.0x or Z51.1 (repetitive treatment with chemotherapy or radiotherapy) associated with codes C00 –C43, C45 – C75, C77 – C79 or C81 – C97 as related diagnosis during second semester. Excluding stays which, after linkage, have a first-semester principal diagnosis of Z51.0x or Z51.1 and a related diagnosis of C00 –C43, C45 – C75, C77 – C79 or C81 – C97.

First patient’s stays during the second semester

Exclusion criteria:

Malignant skin tumors other than C44

Tumors in situ D00 to D09

Tumors with unpredictable or unknown evolution D37 to D48

Malignant tumors with poorly defined localization, secondary and unspecified C76 to C80

Hospital stays with major diagnostic category 28

Hospital stays with error in Diagnosis Related Group

Cancer patient deceased in intensive care unit after surgical act without chemotherapy performed during hospital stay.

Cancer patients hospitalized uniquely for implantation of vascular device and without any chemotherapy performed during hospital stay

**HPI 4: Completeness and Quality of Anesthetic Records**

Inclusion criteria

Hospital stays with activity code (CCAM code) corresponding to 4 (general or loco-regional anesthesia).

Exclusion criteria

Patients hospitalized for intervention under local anesthesia

Hospital stays with major diagnostic category 28

Hospital stays with service inter-establishment

**HPI 5: Completeness and Quality of Medical Records**

Inclusion criteria

Hospital stays of at least one day

Exclusion criteria

Hospital stays with the major diagnostic category 28

Hospital stays with day of entry corresponding to day of discharge

Newborns hospital stays “non-hospitalized”

Hospital stays with service inter-establishment

Hospital stays with error in Diagnosis Related Group

Hospital stays with 23K02Z in Diagnosis Related Group

Hospital stays within an UHCD not followed by stay in intensive care.
