# Supplementary material for: Hospital quality measures: are process indicators associated with hospital standardized mortality ratios in French acute care hospitals?
Source: BMC Health Serv Res. 2017 Aug 22;17:578. doi: 10.1186/s12913-017-2534-3 (PMC5568353; doi:10.1186/s12913-017-2534-3)
Supplement: Supplementary file 2 — Description of the SIMEX method [47–50]. (DOCX 48 kb) [file 12913_2017_2534_MOESM2_ESM.docx]

***Additional file 2:***

The SIMEX method comprises four main steps:

Consider a set of values 0= λ_1_ < λ_2_ < λ_3_ …. < λ_j_

**First step - Simulation**: Generate additional independent measurement error added to the observed HPI value (W). This is a computer-simulated standard normal augmentation of the error variance (U). Contaminated datasets are generated with successively larger measurement error variance. For the j^th^ data set, the total measurement error variance is ∑_u_ + λ_j_ ∑_u_ = (1 + λ_j_) ∑_u_

**Second step – Estimation**: For each contaminated data set generated in the previous step, a Poisson model assessing the association between HPI (W) and each outcome (HSMR) is fitted.

**Third step - Replication**: The first two steps are repeated many times (e.g. 1000). For each level of contamination, the average value of the estimates is calculated. A graph is drawn between the level of contamination values and the estimated average with a quadratic regression. If the quadratic regression does not fit the data well, a linear regression is performed.

**Fourth step - Extrapolation**: Corrected coefficient is estimated with the extrapolation model. The true coefficient corresponds to the ideal case without any measurement error (λ_0_= -1)

**Figure S1**: SIMEX graphic association between BASI Score (HPI2) and 30 dpa HSMR


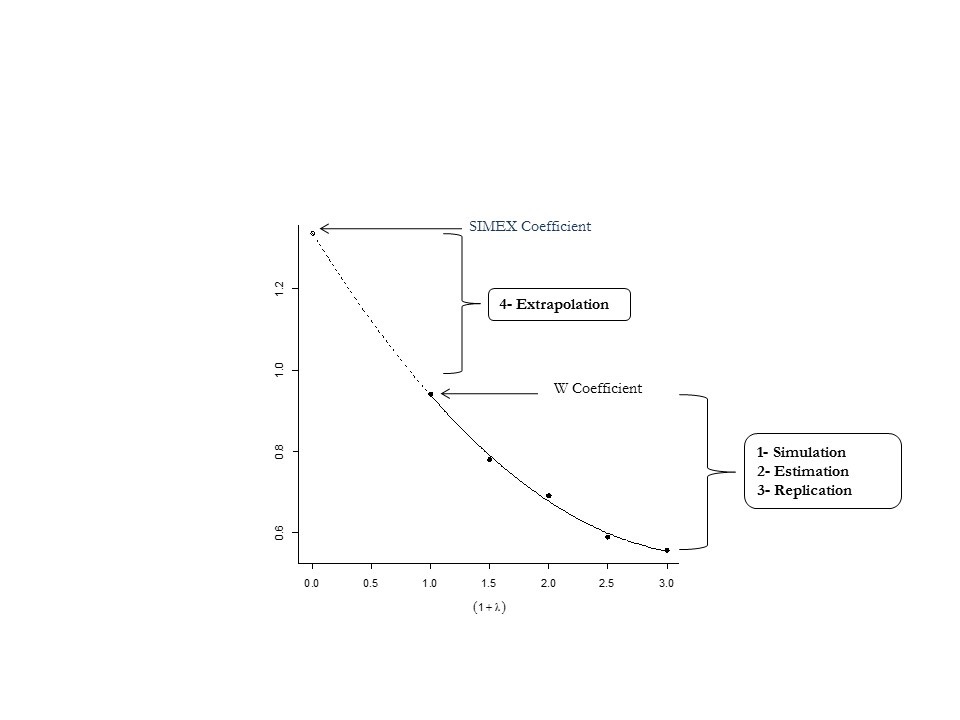


The precision of the corrected coefficient HPI (true coefficient) is estimated using two techniques: the asymptotic method and the jackknife method [47]. The choice depends on the type of variance [48] heteroscedastic (jackknife method) versus homoscedastic (asymptotic method) variance. Heteroscedasticity or homoscedasticity was tested using two known tests: The White [49] and Breusch-Pagan tests [50]. We established a conservative rule whereby if one of the two tests concluded in heteroscedasticity, we used the jackknife method to estimate precision. Otherwise, if neither test concluded in heteroscedasticity, we conclude in homoscedasticity.
